# Drug‐Coated Balloons Versus Non‐Coated Balloons for Side Branch Treatment in Bifurcation Lesions: A Systematic Review and Meta‐Analysis

**DOI:** 10.1002/ccd.31571

**Published:** 2025-05-09

**Authors:** Matteo Rocchetti, Lorenzo Tua, Alberto Cereda, Barbara Conconi, A. Gabriele Franchina, Matteo Carlà, Andrea Spangaro, Stefano Lucreziotti

**Affiliations:** ^1^ Division of Cardiology, Cardio‐Thoracic Department San Carlo Borromeo Hospital (ASST Santi Paolo e Carlo) Milan Italy; ^2^ University of Milan Milan Italy

**Keywords:** ANCO—angiography, BALA—balloon angioplasty, BALD—balloon, BIFL—bifurcation lesions, complex PCI, coronary, DES—stent, drug coated/eluting, drug eluting, PCIC—percutaneous coronary intervention

## Abstract

**Background:**

Coronary bifurcation lesions (CBL) account for ~20% of percutaneous coronary interventions (PCI). When using provisional stenting, optimal management of a diseased side branch (SB) remains debated. Drug‐coated balloons (DCBs) are an emerging option, but data on their role in CBL PCI are limited.

**Aims:**

We conducted a meta‐analysis to compare DCBs and non‐compliant balloons (NCBs) for SB treatment.

**Methods:**

A systematic search of MEDLINE, CENTRAL, and EmBase (until November 2024) identified studies comparing DCBs and NCBs for treating the SB. From 1451 studies, five were included (two randomized controlled trials [RCTs], three observational), with 1762 patients, predominantly male with acute coronary syndrome (ACS). The primary outcome was major adverse cardiac events (MACE); secondary outcomes included myocardial infarction (MI), target lesion revascularization (TLR), and target vessel revascularization (TVR).

**Results:**

DCBs significantly reduced MACE (pooled OR 0.48, 95% CI 0.34–0.68, *p* < 0.0001, *I*² = 0%) and MI (pooled OR 0.39, 95% CI 0.25–0.62, *p* < 0.001, *I*² = 0%) compared to NCBs. No significant differences were observed in TLR or TVR. Subgroup analysis confirmed consistency across observational studies and RCTs for MACE and MI.

**Conclusions:**

DCBs in SB treatment during CBL PCI are associated with reduced MACE and MI compared to NCBs, with no significant differences in vessel‐specific outcomes. These findings suggest a potential clinical benefit of DCBs in reducing ischemic events, while limiting stenting in CBL. Further research is needed to refine patient selection and optimize outcomes.

## 1 Introduction

Coronary bifurcations lesions (CBL) represent a significant challenge in percutaneous coronary intervention (PCI), accounting for 15%−20% of all PCIs [1]. The first step is always represented by the choice between an upfront 2‐stents strategy versus a single‐stent strategy. Provisional stenting usually represents the default technique for the treatment of most CBL [1]. However side branch (SB) compromise after main vessel (MV) stenting occurs in 6%−18% of cases [2], mainly due to carina shift or overexpansion of the distal part of the MV stent. Dilatation of the compromised SB with a non‐coated balloon (NCB) is often sufficient for restoring the vessel's patency, without the need of additional stents. In the unfortunate scenario of a bailout 2‐stent strategy, the new carina, completely covered in metal, represents a fertile environment for both thrombosis and in‐stent restenosis [3, 4]. Thus, an enthusiastic interest has grown around the possibility of using drug‐coated balloons (DCBs) in the treatment of SB to minimize the stent‐covered surface. DCBs are covered with specific drug‐coatings with an anti‐intimal hyperplasia effect rapidly absorbed by vascular wall tissue [5]. The most common drug coating is paclitaxel, which reduces cell differentiation and the release of platelet‐derived growth factors and inhibits the migration of vascular smooth muscle cells to the intima following the inflammatory response that is triggered by any balloon inflation [6]. While the role of DCBs in treating in‐stent restenosis and small coronary arteries has been investigated and widely recognized [7, 8], clinical benefits of DCBs compared to NCBs for the treatment of SB following stenting of the MV in true CBL still represent a matter of debate, given the conflicting results from randomized and non‐randomized trials

Given this fertile field of research and a recently published randomized controlled trial (RCT), we performed a meta‐analysis of clinical studies to systematically evaluate and compare the effectiveness and safety of DCBs versus NCBs in the treatment of SB, following provisional stenting of the MV, focusing on clinical outcomes such as major adverse cardiac events (MACE), myocardial infarction (MI), target lesion revascularization (TLR) and target vessel revascularization (TVR) (Central Illustration 1).

## 2 Methods

### 2.1 Search Strategy

Until November 2024, we systematically searched MEDLINE, Cochrane Central Register of Controlled Trials (CENTRAL), and EmBase databases for relevant studies. Terms used to find the desired articles were “bifurcation lesion” and “drug‐coated balloon,” along with relevant Medical Subject Headings (MeSH) terms and Emtree terms derived from these keywords to ensure comprehensive coverage of the topic. The search only included peer‐reviewed studies on human subjects published in English language.

### 2.2 Study Selection

**Figure 1:**
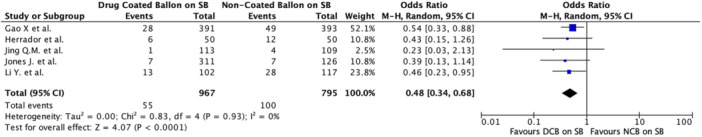
Major adverse cardiovascular events (MACE) displayed for both randomized clinical trial and observational studies. DCB, drug coated balloon; NCB, non‐coated balloon; SB, side branch. [Color figure can be viewed at wileyonlinelibrary.com]

The research initially yielded 1451 total results, of which 1003 results from MEDLINE, 148 results from EmBase, and 300 results from the CENTRAL. After removing duplicates, a total of 1087 articles were screened based on their titles. Subsequently, 109 articles underwent abstract screening, and 21 articles were assessed for eligibility through full‐text review. Finally, five articles were selected for inclusion in this meta‐analysis (Figure 1: Supporting Information Materials S1). We included both RCTs and observational retrospective studies. Studies were considered eligible if they directly compared DCBs and NCBs in the SB for the treatment of bifurcation lesions using the provisional technique. Conversely, studies evaluating the DCB‐alone strategy, where DCBs were used in both the MB and SB, were excluded from the analysis. Only articles reporting at least one clinical endpoint of interest (see below) were selected.

Two independent reviewers (M.R. and L.T.) conducted the screening process and assessed the eligibility of studies according to predefined inclusion and exclusion criteria. Discrepancies between the reviewers were resolved through discussion or consultation with a third reviewer. The Kappa statistic was utilized to assess the inter‐rater reliability of the two reviewers [9].

### 2.3 Data Extraction

Two authors (M.R. and L.T.) extracted inherent data independently using a standardized recording tool to document the study setting and design, year of publication, number of study participants, country of origin, participant clinical characteristics (age, gender, and cardiovascular risk factor), clinical presentation between acute coronary syndrome (ACS) or chronic coronary syndrome (CCS), bifurcation type according to the Medina classification [10], diseased coronary artery, previous MI and study outcomes.

### 2.4 Identified Outcomes

The primary outcome we investigated was MACE, as defined by each study. Secondary outcomes included MI, TVR, and TLR where available. Due to the low number of events for TVR and TLR in the included studies, a composite endpoint termed “treated‐vessel specific endpoint” (TVSE) was created. TVSE was defined as the sum of TVR and/or TLR events, allowing for a more meaningful analysis of outcomes related to the treated vessel.

### 2.5 Statistical Analysis

Quantitative data are reported as mean values with standard deviation, and categorical variables are expressed in terms of percentages. Statistical analysis was performed using the Mantel‐Haenszel method for pooled effect size calculation. A random‐effects model was employed due to the heterogeneity across studies. Heterogeneity was assessed using the *I*² statistic, with values of 25%, 50%, and 75% representing low, moderate, and high heterogeneity, respectively. Statistical significance was defined as *p* < 0.05. To assess potential publication bias, we utilized funnel plots (Supporting Information Materials). Risk of bias (RoB) for each included study was evaluated by two reviewers using Cochrane RoB 2 for randomized trial and Newcastle Ottawa scale tools for observational trial (Supporting Information Materials Tables 1.1, 1.2, and 1.3). All statistics were calculated both considering RCTs and observational retrospective studies separately, as well as combining the two types of studies to provide a comprehensive analysis. Meta‐regression was conducted to explore sources of heterogeneity using the extracted parameters (e.g., patient characteristics, lesion complexity, and procedural details).

### 2.6 Registration and Reporting Standards

This meta‐analysis was prospectively registered on the PROSPERO database to ensure transparency and adherence to systematic review and meta‐analysis guidelines (ID CRD42025632460). The study was conducted and presented according to best practice recommendations, including the Preferred Reporting Items for Systematic Reviews and Meta‐Analyses (PRISMA) reporting guidelines [11].

## 3 Results

A total of five studies, comprising 1762 patients, were included in this meta‐analysis (Table 1). The studies consisted of two RCTs [12, 13] and three observational studies [14, 15, 16], with sample sizes ranging from 100 to 784 participants. The two RCTs were conducted primarily in hospitals located in China, Italy, Indonesia, and the Republic of Korea, while the three observational studies predominantly recruited patients from China, Spain, and the United Kingdom.

**Table 1:** Summary of studies included in the meta‐analysis. DCBs, drug‐coated ballons; NCB, non‐coated balloons.

Author(s)	Year	Country	Sample Size	Study type	DCBs (*n*)	NCBs (*n*)
Gao et al. [12]	2024	China, Italy, Indonesia and Republic of Korea	784	Randomized Clinical Trial	391	393
Jing et al. [13]	2020	China	222	Randomized Clinical trial	113	109
Herrador et al. [14]	2013	Spain	100	Observational	50	50
Jones et al. [15]	2023	United Kingdom	437	Observational	311	126
Li et al. [16]	2021	China	219	Observational	102	117

The proportion of males ranged from 73% to 83% across the studies. Cardiovascular risk factors were common in all studies, with diabetes affecting 30%− 37% of participants, dyslipidemia 23%−65%, smoking history 40%−53%, and hypertension 52% to 64%. The proportion of patients presenting with ACS ranged from 56% to 91%, while those with CCS ranged from 4% to 24%.

The type of CBL according to the Medina classification differed substantially among the included studies but always included “true” bifurcations (i.e., Medina 1,1,1 or 1,0,1 or 0,1,1). The prevalence of each subtype in each study can be found in the Supporting Information Materials Table 1.2, Table 2 overall, the most prevalent were the 1,1,1 bifurcations. Regarding the technique for SB PCI, Gao et al. and Jones et al. were the only studies that explicitly described the use of the kissing balloon (KB) technique [12, 15].

**Table 2:** Summary of main characteristics of populations included in the meta‐analysis. The percentages refer to the whole population included in each study (both intervention and control arm).

Author(s)	Males (%)	Diabetes (%)	Dyslipidemia (%)	Smoking (%)	Hypertension (%)	ACS (%)	CCS (%)	Medina 1;1;1 (%)	LAD (%)
Gao et al. [12]	77%	37%	62%	40%	64%	91%	9%	68%	77%
Jing et al. [13]	73%	32%	23%	54%	60%	92%	4%	77%	73%
Herrador et al. [14]	83%	33%	56%	52%	62%	76%	24%	N.A.	83%
Jones et al. [15]	76%	31%	65%	53%	52%	56%	28%	57%	76%
Li et al. [16]	81%	30%	24%	55%	53%	N.A.	N.A.	28%	81%

Abbreviations: ACS, acute coronary syndrome; CCS, chronic coronary syndrome; LAD, left anterior descending artery.

**Figure 2:**
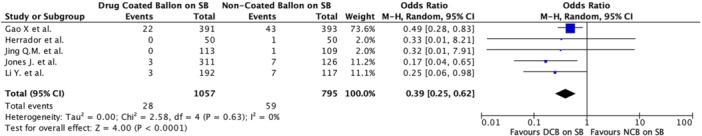
Myocardial infarction (MI) according to both randomized clinical trial and observational trial. DCB, drug coated balloon; NCB, non‐coated balloon; SB, side branch. [Color figure can be viewed at wileyonlinelibrary.com]

**Figure 3:**
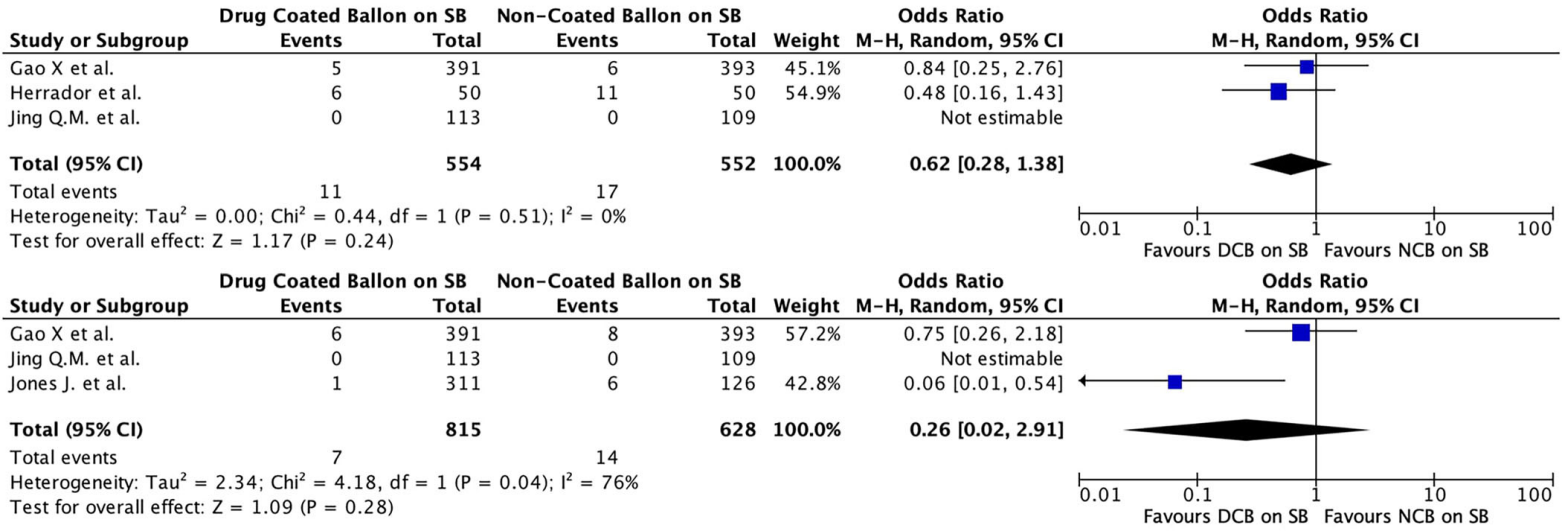
Forest plot displaying target lesion revascularization (TLR), above, and target vessel revascularization (TVR), below, for randomized clinical trial and observational trial combined. DCB, drug coated balloon; NCB, non‐coated balloon; SB, side branch. [Color figure can be viewed at wileyonlinelibrary.com]

**Figure 4:**
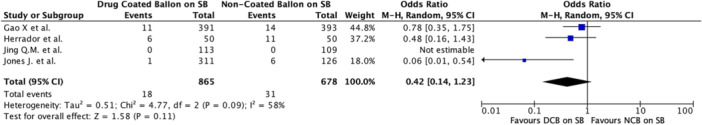
Forest plot showing the combined endpoint “target specific vessel endpoint” (TSVE: TLR + TVR) for randomized clinical trial and observational trial combined. DCB, drug coated balloon; NCB, non‐coated balloon; SB, side branch. [Color figure can be viewed at wileyonlinelibrary.com]

Regarding the primary outcome (MACE), PCI with DCBs in the SB was associated with a significantly lower risk compared to NCBs. The pooled odds ratio (OR) for MACE was 0.48 (95% CI 0.34–0.68, *p* < 0.0001), with negligible heterogeneity (*I*² = 0%), indicating strong consistency in the results across the included studies (Table 3 and Figure 1). In terms of secondary outcomes, MI was significantly reduced in the DCB group, with a pooled OR of 0.39 (95% CI 0.25–0.62, *p* < 0.001) and negligible heterogeneity (*I*² = 0%) (Figure 2). The analysis for TLR across studies showed an OR of 0.62 (95% CI 0.28–1.38), with no significant difference between the DCB and NCB groups (*p* = 0.24) albeit low heterogeneity (*I*² = 10%). The same applied for TVR, with pooled OR 0.26 (95% CI 0.02–2.91, *p* = 0.28) and moderate heterogeneity (*I*² = 76%) (Figure 3). Finally, regarding the composite TVSE endpoint, the pooled OR was 0.42 (95% CI 0.14–1.23, *p* = 0.11), with moderate heterogeneity (*I*² = 58%) (Figure 4).

**Table 3:** Summary of main events in each included study.

Author(s)	MACE (DCB)	MACE (NCB)	TLR (DCB)	TLR (NCB)	TVR (DCB)	TVR (NCB)	MI (DCB)	MI (NCB)	TVSE (DCB)	TVSE (NCB)
Gao et al. [12]	28	49	5	6	6	8	22	43	11	14
Jing et al. [13]	1	4	0	0	0	0	0	1	0	0
Herrador et al. [14]	6	12	6	11	—	—	0	1	6	11
Jones et al. [15]	7	7	—	—	1	6	3	7	1	6
Li et al. [16]	13	28	—	—	—	—	3	7	—	—

Abbreviations: DCB, drug‐coated balloon; MACE, Major Adverse Cardiovascular Event; MI, myocardial infarction; N.A., not available; NCB, non‐coated balloon; TLR, Target Lesion Revascularization; TVR, Target Vessel revascularization; TVSE, target vessel specific endpoint.

**Figure 5:**
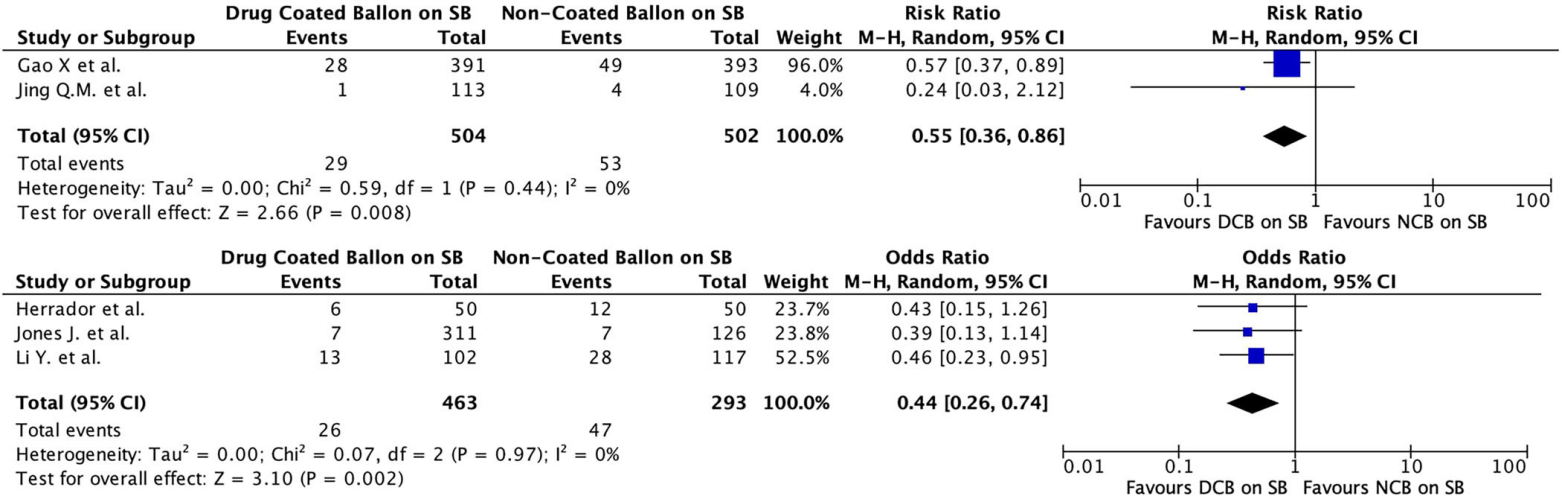
Subgroup analysis for major adverse cardiovascular events (MACE) divided according to study design (randomized clinical trial above, observational study below). [Color figure can be viewed at wileyonlinelibrary.com]

Subgroup analyses provided additional insights: observational studies showed a significant OR for MACE (pooled OR 0.44, 95% CI 0.26–0.74, *p* = 0.002) with low heterogeneity (*I*² = 15%). RCT also demonstrated an OR of 0.52 (95% CI 0.32−0.84) for MACE (*p* = 0.007). These findings underscore the consistency of the benefits of DCBs in both prospective and retrospective studies analyzed separately (Figure 5).

**Figure 6:**
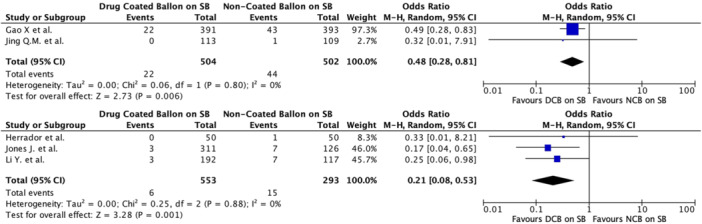
Subgroup analysis of myocardial infarction (MI) randomized clinical trial (above) and observational trial (below). [Color figure can be viewed at wileyonlinelibrary.com]

**Figure 7:**
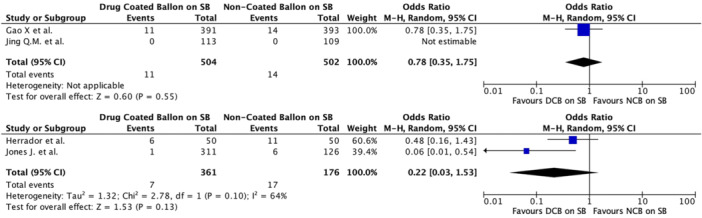
Subgroup analysis of target vessel specific endpoint (TVSE) for randomized clinical trial (above) and observational studies (below). [Color figure can be viewed at wileyonlinelibrary.com]

The results of subgroup analyses examining MI in RCTs showed a pooled OR of 0.48 (95% CI 0.28–0.81, *p* = 0.006) with negligible heterogeneity (*I*² = 0%). Similarly, observational studies showed a pooled OR for MI of 0.21 (95% CI 0.08–0.54, *p* = 0.001) and negligible heterogeneity (*I*² = 0%) (Figure 6). Subgroup analyses were also performed for TVSE (Figure 7), without providing any additional significant information, while no subgroup analysis for TVR or TLR was conducted due to insufficient data across the included studies.

Meta‐regression on baseline patient characteristics, lesion complexity, and procedural details, revealed female sex as the only significant source of heterogeneity among studies (*p* > 0.001).

**Central Illustration 1:**
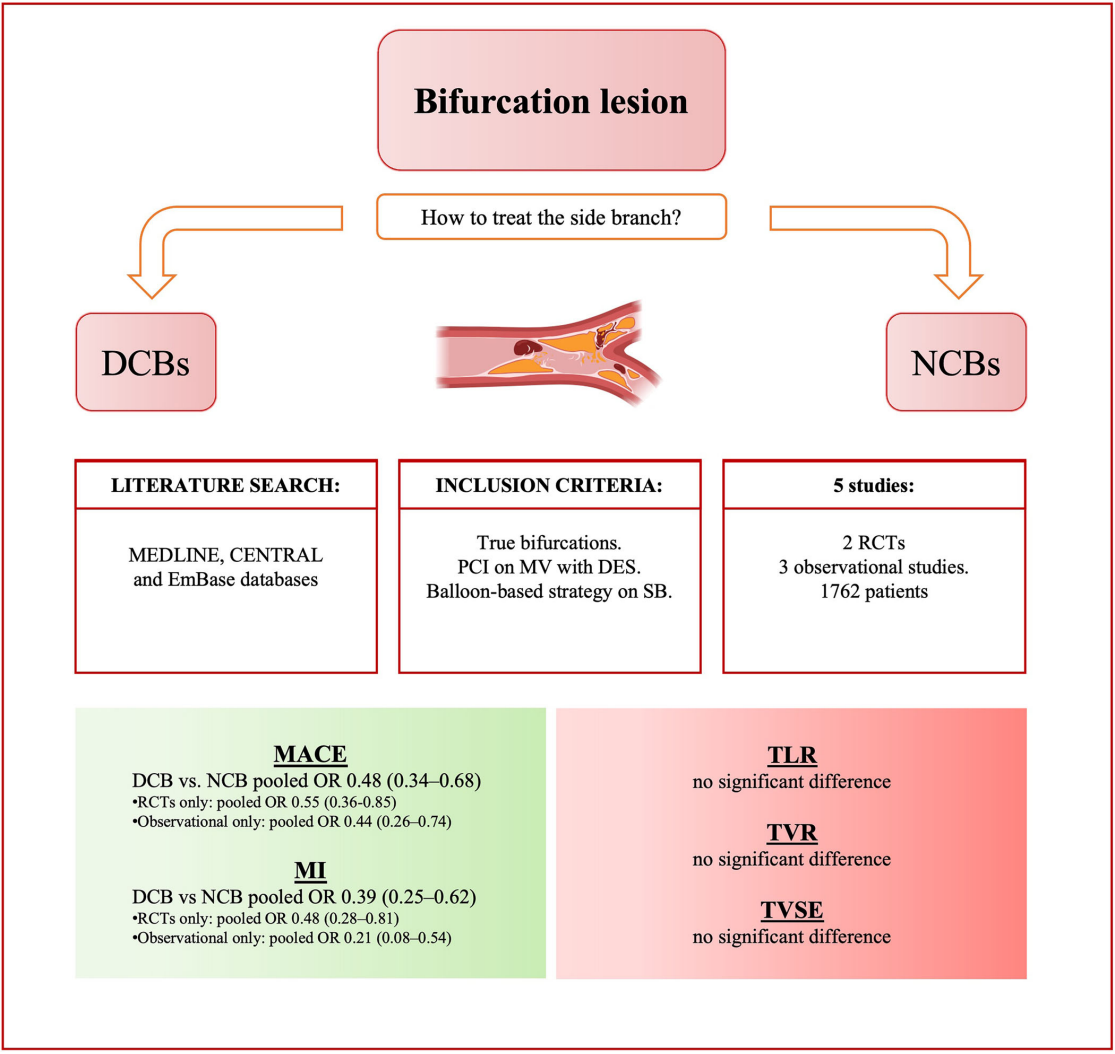
DCBs, drug‐coated balloons; MACE, major adverse cardiovascular events; MI, myocardial infarction; MV, main vessel; NCBs, non‐coated balloons; OR, odds ratio; PCI, percutaneous coronary intervention; RCTs, randomized controlled trials; SB, side branch: TLR, target lesion revascularization; TVR, target vessel revascularization; TVSE, target vessel specific endpoint. [Color figure can be viewed at wileyonlinelibrary.com]

## 4 Discussion

This meta‐analysis builds upon previous studies by incorporating the most recent evidence, including the recent DCB‐BIF trial [12], refining the inclusion criteria, and ensuring alignment with contemporary clinical practice.

Our work provides important insights into the use of DCBs compared to NCBs on the SB for the treatment of CBL. Overall, the results of this meta‐analysis showed a significant benefit of DCBs in reducing MACE and MI compared to NCBs. In contrast, outcomes such as TLR, TVR, and TVSE did not achieve statistical significance, although trends favoring DCBs were observed. Regarding these vessel‐specific outcomes, the lack of significance may be attributable to the relatively low number of events, leading to limited statistical power. Furthermore, the moderate heterogeneity observed in TVR (*I*² = 76%) and TVSE (*I*² = 55%) suggests variability across studies, which may have influenced these results.

The importance of maintaining SB patency during provisional stenting has been addressed in several consensus papers, given its fundamental role in any CBL PCI [1, 17]. A “relevant” SB is defined as one with a 2.0 mm diameter that supplies at least 10% of myocardial mass; a SB length of 73 mm has been suggested as a surrogate for the mass parameter [18]. Predisposing factors for SB occlusion after MV include spiky carina morphology (“eyebrow sign”), a narrow carina tip angle, and calcified plaques, leading to the development of risk stratification scores [19, 20, 21, 22]. The most widely used is the RESOLVE score, which comprises 6 independent variables and a score ≥ 10 predicts a 20% chance of SB occlusion [21]. Whatever the preferred technique to ensure SB patency is adopted (i.e., “jailed wire technique,” “jailed balloon technique,” etc…) [1], in the unfortunate event the SB is compromised, the preferred approach is to restore patency and flow with a balloon. Traditionally, a NCB was used to open the stent struts toward the SB, followed by a KB in the MV with another NCB. With the advent of DCBs, interest grew in treating compromised SBs without extra stents while delivering anti‐proliferative drugs. Until the DCB‐BIF trial, several studies addressed this topic, but were mainly flawed by small sample size, varying follow‐up duration and pre‐defined outcomes, which led to conflicting results [12]. Similarly, between the two included RCTs in our meta‐analysis, the DCB‐BIF Trial (784 patients, the majority with unstable angina—61.1%—followed by NSTEMI—24.5%—) was the first large multicenter RCT comparing DCBs and NCBs in the SB [12] after MV provisional stenting. The trial exclusively included true bifurcation lesions according to the Medina classification, with simple SB involvement, defined as a lesion length of < 10 mm, according to the DEFINITION (Definitions and Impact of Complex Bifurcation Lesions on Clinical Outcomes After PCI Using Drug‐Eluting Stents) criteria [4]. The provisional stenting strategy was performed in alignment with the European Bifurcation Club (EBC) guidelines [1], and routine pre‐dilatation of the SB was generally avoided. The trial showed significant reductions in MACE and MI in patients treated with DCBs, possibly driving the main results of our meta‐analysis. In contrast, the second RCT, the BEYOND study, was a much smaller trial (222 patients with nearly 91% ACS) which did not show significant difference in MACCEs, non‐fatal MI, TLR and TVR [13]. Low event rates—5 MACCEs (4 in NCB, 1 in DCB), 1 MI (NCB), and no TLR/TVR—combined with a small sample size likely explain the differing RCT results. Additionally, the unblinded design, variations in reference vessel minimal lumen diameter, and exclusion of distal left main bifurcations may account for BEYOND's negative DCB versus NCB findings.

RoB assessments revealed notable differences between the two RCTs. The larger, recent DCB‐BIF trial had low bias—with strong randomization and blinding of patients and outcome assessors, aside from operator blinding. In contrast, BEYOND had higher bias due to 21% missing angiographic follow‐up at 9 months, reliance on LOCF imputation, and unclear blinding. These quality differences may explain the trials' discrepant findings, while the robust DCB‐BIF trial supports the benefits of DCBs over NCBs in SB treatment.

Previously, some meta‐analyses have addressed the use of DCBs for the treatment of SB in CBL [23, 24, 25, 26]. While all concluded that DCB improves clinical outcomes compared to NCBs, there are important methodological differences between these analyses and our study. Notably, three of these meta‐analyses [23, 24, 25] included studies where the MV was either untreated or treated with bare‐metal stents (BMS). This possibly reflects the absence of significant lesions in the MV, thus differing from the true bifurcations we included in our analysis, while the use of BMS represents nowadays an outdated practice. Additionally, two meta‐analyses [25, 26], included a larger number of studies by considering numerous articles in Chinese language, not easily accessible for the international community.

Our study focused on a common interventional cardiology scenario—provisional stenting complicated by SB compromise. We selected studies where the MV was stented with DES and subsequent measures restored SB flow, avoiding a bailout second stent.

Subgroup analyses add context: RCTs showed a significant MACE reduction (OR 0.48, 95% CI 0.34–0.68, *p* < 0.0001, *I*² = 0%), matching the strong reduction in observational studies (OR 0.21, 95% CI 0.08–0.54, *p* = 0.001, *I*² = 0%). This consistency supports DCB efficacy across study designs, with RCTs providing rigor and observational studies offering real‐world insights.

The findings of this analysis should be interpreted considering several limitations. First, the inclusion of both prospective and retrospective studies introduces variability in study design, patient selection, and data quality. Second, the limited number of included studies and, thus, the often‐low number of events, particularly for secondary endpoints, reduces the statistical power to detect significant differences. Third, the results are largely driven by the DCB‐BIF Trial, which is the largest and most robust RCT included. While its findings are consistent with other studies, the influence of a single large trial can skew the overall results, particularly when the remaining studies have smaller sample sizes and fewer events. This emphasizes the need for additional large‐scale RCTs to validate the findings and ensure broader applicability across different patient populations. Furthermore, the variability in procedural strategies across studies, such as the KB technique described only in Gao et al [12] and Jones et al. [15], and the provisional T‐stenting technique reported by Herrador et al. [14], introduces an additional layer of heterogeneity. The lack of detailed procedural descriptions in other studies limits our ability to fully assess the impact of these techniques on clinical outcomes. Among the two RCTs included, only the DCB‐BIF trial reported data on crossover to DES in the SB for both the DCB and NCB groups. In this study, no significant difference was observed in crossover rates between the two populations. SB size data varied greatly across studies, preventing a unified analysis to identify a specific diameter favoring DCB benefit. However, meta‐regression showed that studies with more female patients tended to favor a DCB strategy—suggesting that, since women usually have smaller coronary arteries, they might derive greater benefit from DCBs. Finally, publication bias cannot be entirely excluded, although funnel plot analysis did not reveal significant asymmetry.

Despite its limitations, this meta‐analysis shows that using DCBs on the SB can improve outcomes in CBL patients treated with DES in the MV. DCBs provide a clear advantage over NCBs—avoiding extra stent implantation and yielding better outcomes than plain balloon angioplasty. This is particularly valuable when SB treatment is unplanned, such as in bailout PCI after provisional stenting in a single‐stent strategy.

Future research should aim to address the limitations of the current analysis with large, multicenter RCTs, longer follow‐up durations and subgroup analyses exploring the impact of lesion characteristics, procedural techniques, and adjunctive therapies with the aim of optimizing clinical outcomes and reduce mortality.

## 5 Conclusion

This meta‐analysis suggests that the use of DCBs in the treatment of SB during PCI for CBL is associated with less MACE and MI compared to NCBs. No significant difference was found regarding vessel‐specific outcomes (i.e., TLR or TVR), possibly due to the low number of events of the selected studies, although a trend in favor of DCB was noted. The results of this study strengthen the evidence around the use of DCBs as an effective alternative to NCBs, particularly in scenarios where minimizing additional stent implantation in the SB is desirable. Furthermore, these findings also align with current clinical practice favoring, whenever possible, a single‐stent approach and reserving the treatment of the SB to a subsequent evaluation of its potential compromise. This study provides a strong foundation for further exploration of DCBs in more complex coronary artery disease settings. Future research should also focus on standardizing procedural strategies to ensure consistent application of DCBs in bifurcation PCI.

## Conflicts of Interest

The authors declare no conflicts of interest.

## Supporting information

Supporting Material.

## Data Availability

The data that support the findings of this study are available from the corresponding author upon reasonable request.
