# Supplementary material for: Drug‐Coated Balloons Versus Non‐Coated Balloons for Side Branch Treatment in Bifurcation Lesions: A Systematic Review and Meta‐Analysis
Source: Catheter Cardiovasc Interv. 2025 May 9;106(1):530–9. doi: 10.1002/ccd.31571 (PMC12231156; doi:10.1002/ccd.31571)

Supplementary materials

Summary

[Search strategy 1](#_Toc192457093)

[Risk of bias 2](#_Toc192457094)

[Patients caratheristics 3](#_Toc192457095)

[Meta regression analysis 4](#_Toc192457096)

[Funnel plots 5](#_Toc192457097)

# Search strategy

**Identification of studies via databases and registers**

Records removed *before screening*:

Duplicate records removed (n = 425)

Records identified from:

Databases (n =1)

Registers (n = 1512)

**Identification**

Records excluded

(n =978)

Records screened based on title

(n =1087)

Reports excluded

(n =88)

Reports screened based on abstract

(n =109)

**Screening**

Reports excluded:

(n=16)

Reports assessed for eligibility

(n = 21)

Randomized studies included

(n = 2)

Observational studies included

(n = 3)

**Included**

**Figure 1:** PRISMA flow diagram for study selection

Source : Page MJ, et al. BMJ 2021;372:n71. doi: 10.1136/bmj.n71.

# Risk of bias

| Selection | | | | | Comparability | | | Outcome | | Total |
| --- | --- | --- | --- | --- | --- | --- | --- | --- | --- | --- |
|  | Representativeness of the exposed cohort | Selection of the non exposed cohort | Ascertainment of exposure | Demonstration that outcome of interest was not present at start of study | Main factor | Additional factor | Assessment of outcome | Was follow-up long enough for outcomes to occur | Adequacy of follow up of cohorts |  |
| Herrador A.J. et al. | + | + | + | + | + | + | + | + | \ | 8\9 |
| Jones J et al. | + | + | + | + | \ | \ | + | + | + | 7\9 |
| Li Y. et al. | + | + | + | + | + | + | + | + | + | 9\9 |
| Table 1.1 Risk of bias for observational trials, New Castle Ottawa Scale | | | | | | | | | | |

| Domain | Risk of Bias Judgment | Justification |
| --- | --- | --- |
| Bias arising from the randomization process | Low risk | The study used an interactive web-based system with permuted block randomization, ensuring proper allocation concealment and reducing selection bias. No major concerns regarding baseline imbalances were reported. |
| Bias due to deviations from the intended interventions | Some concerns | Blinding was not possible for operators in the cath lab, which could introduce performance bias. However, patients, treating physicians, and outcome assessors were blinded, reducing detection bias. |
| Bias due to missing outcome data | Low risk | There was minimal loss to follow-up (770 per protocol vs. 784 ITT), and missing data were not imputed, but this small difference is unlikely to meaningfully affect the results. |
| Bias in measurement of the outcome | Low risk | The study reported objective clinical endpoints (MACE, MI, TLR, TVR, etc.), reducing the likelihood of measurement bias. |
| Bias in selection of the reported result | Low risk | All pre-specified outcomes were reported in the study, with no evidence of selective outcome reporting. |
| Overall Risk of Bias Judgment: Some concerns (due to lack of operator blinding), but overall low risk of bias in key domains, making the results reliable. | | |
| Table 1.2 Risk of Bias Assessment (RoB2) for DCB-BIF Trial | | |

| Domain | Risk of Bias Judgment | Justification |
| --- | --- | --- |
| Bias arising from the randomization process | Some concerns | The study used block randomization (block size of 4) by center with sealed, opaque envelopes, which ensures some level of allocation concealment. However, the method carries a potential risk of predictability, particularly in smaller trials. There was an umbalance in the percentage of males patients in the DCB group. |
| Bias due to deviations from the intended interventions | Some concerns | Due to the nature of the procedure, clinicians were not blinded. The article does not explicitly state whether patients, outcome assessors, or other key figures were blinded, which increases the risk of performance and detection bias. |
| Bias due to missing outcome data | Some risk | The study had one lost to follow-up, but 47 patients (21%) refused angiographic follow-up at 270 days. While missing data for the primary endpoint were imputed using the last observation carried forward (LOCF) method, this approach assumes no further change in vessel stenosis, which may not be realistic. The high proportion of missing angiographic data raises concerns about potential bias in outcome assessment. |
| Bias in measurement of the outcome | Some concerns | The study used quantitative coronary angiography for the primary endpoint (target lesion stenosis at 9 months), which is an objective measurement. However, clinical outcomes were assessed by outpatient visits or telephone follow-ups, which may introduce inconsistencies in data collection. The lack of a clear blinding process for outcome assessors adds to the concern. |
| Bias in selection of the reported result | Low risk | All pre-specified endpoints were reported in the final publication, with no indication of selective reporting bias. |
| Overall Risk of Bias Judgment: High risk of bias due to significant missing data (21% missing angiographic follow-up) and the uncertainty regarding blinding of patients and outcome assessors. | | |
| Table 1.3: Risk of Bias Assessment (RoB2) for BEYOND Trial | | |

# Patients caratheristics

| Study | DCB group, n (%) | NCB group, n (%) | Total number of patients, n (%) |
| --- | --- | --- | --- |
| Gao X. et al |  | | **784 (100)** |
| Medina classification | | | |
| 1,0,1 | 25 (3.2) | 16 (2) | 41 (5.2) |
| 0,1,1 | 73 (9.3) | 63 (8) | 136 (17.3) |
| 1,1,1 | 293 (37.4) | 304 (38.8) | 597 (76.1) |
| Herrador J. et al |  | | 100 (100) |
| Medina classification | N.A. | |  |
| Jones J. et al. |  | | **437 (100)** |
| Medina Classification | | | |
| 1,0,1 | 8 (1.8) | 17 (3.9) | 25 (5.7) |
| 0,1,1 | 35 (8) | 16 (3.7) | 51 (11.7) |
| 1,1,1, | 199 (45.5) | 66 (15.1) | 265 (60.6) |
| Li Y. et al |  | | 219 (100) |
| Medina classification | | | |
| 1,0,1 | 58 (26.5) | 86 (39.3) | 144 (65.8) |
| 0,1,1 | 17 (7.8) | 10 (4.6) | 27 (12.3) |
| 1,1,1 | 27 (12.3) | 21 (9.6) | 48 (21.9) |
| Quan-Min J. et al |  |  | 222 (100) |
| Medina classification | | | |
| 1,0,1 | 5 (2.3) | 3 (1.4) | 8 (3.6) |
| 0,1,1 | 7 (3.2) | 3 (1.4) | 10 (4.5) |
| 1,1,1 | 101 (45.5) | 101 (45.5) | 202 (91) |
| Table 2. Medina classification of true bifurcations and prevalence of each subtype in each study. DCB: drug coated balloon; N.A., not available; NCB: non-coated balloon. | | | |

# Meta regression analysis


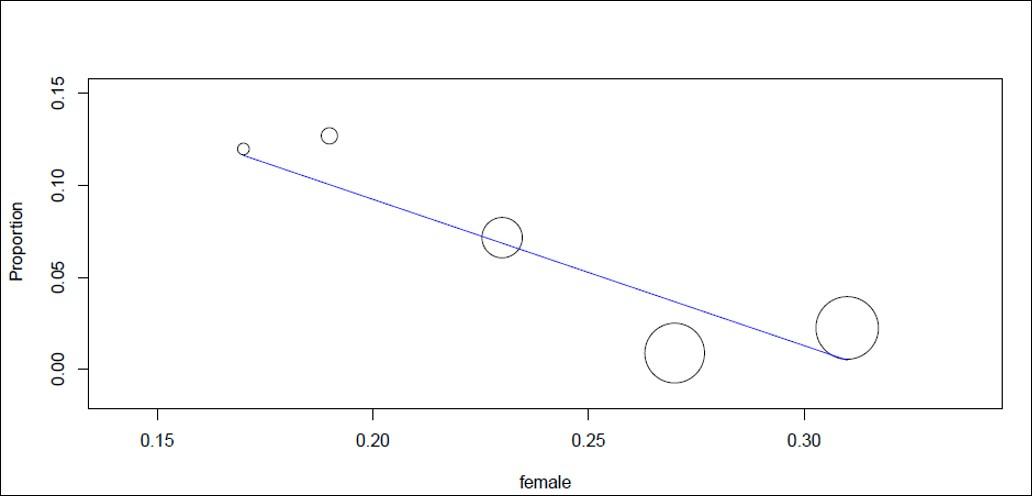


# Funnel plots


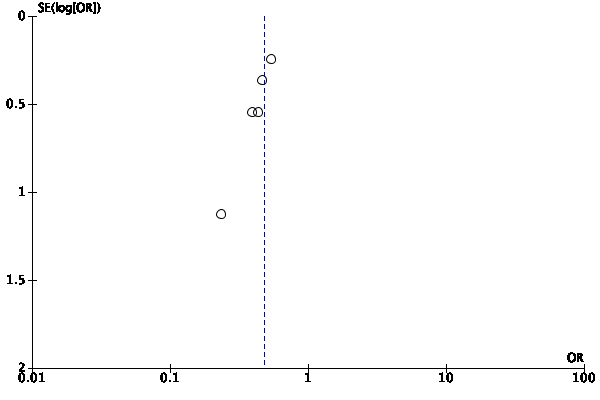

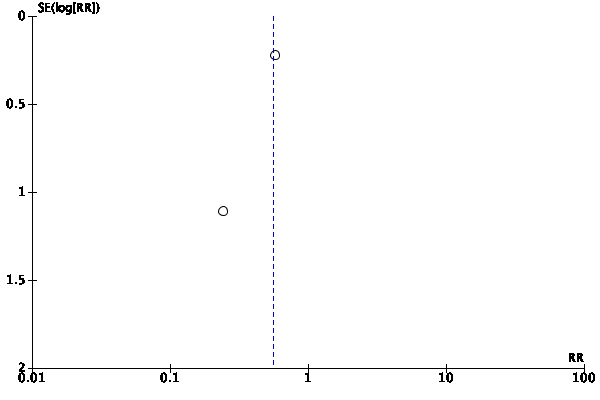

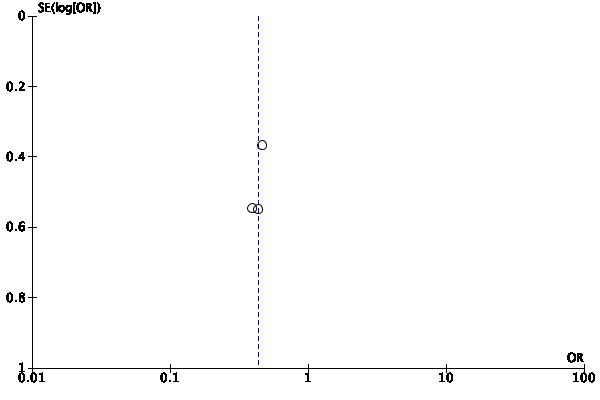


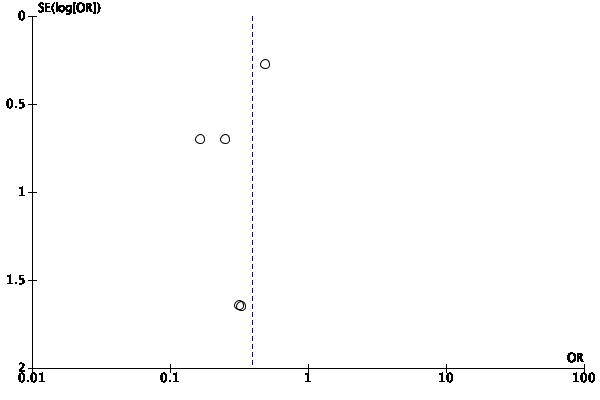

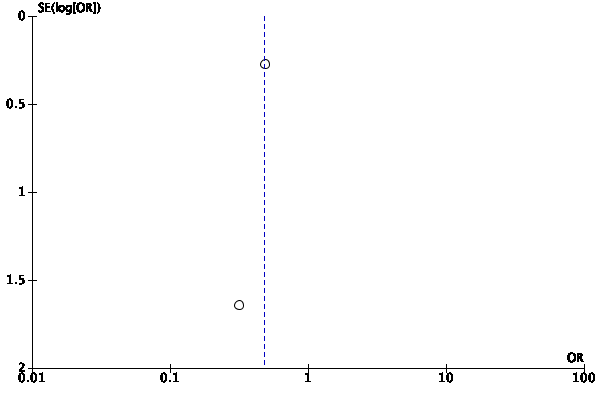

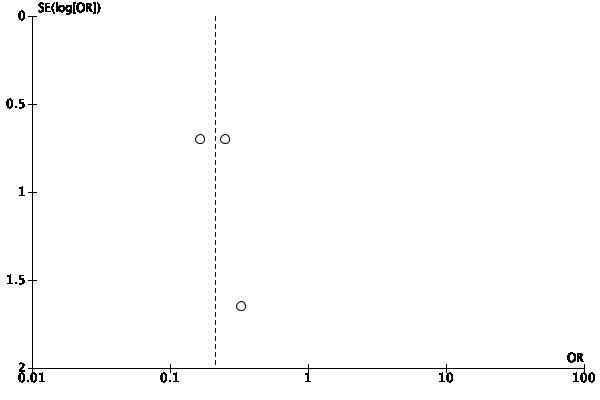


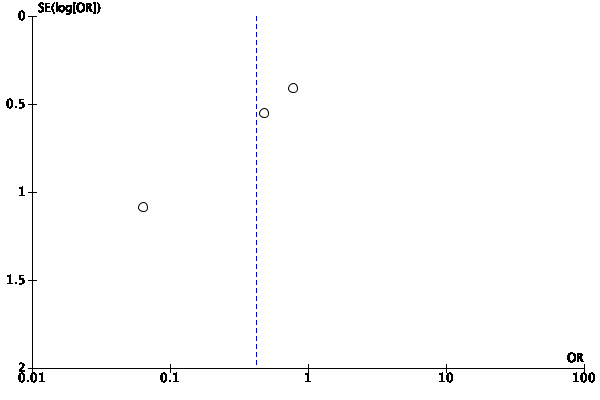

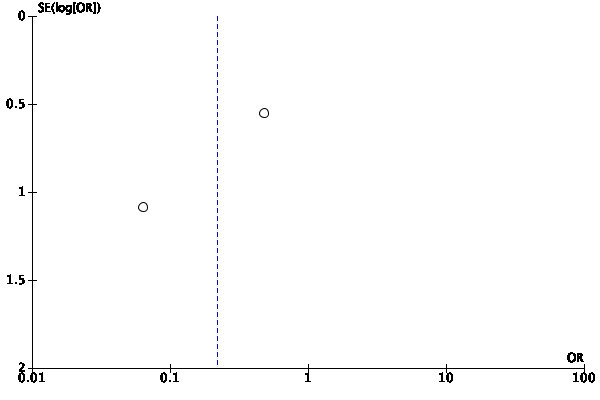

Supplement: Supplementary file 1 — Supporting Material. [file CCD-106-530-s001.docx]
